# Building a framework for reproducibility: the case for standardized data reporting and metadata integration in zebrafish research

**DOI:** 10.1242/dmm.052441

**Published:** 2025-11-18

**Authors:** Mee S. Ngu, Sabrina Toro, Alexa Burger, Khai C. Ang

**Affiliations:** ^1^ Department of Pathology, Penn State College of Medicine, Hershey, PA 17033, USA; ^2^ Department of Genetics, School of Medicine, University of North Carolina at Chapel Hill, Chapel Hill, NC 27599-7264, USA; ^3^ Department of Orthopedics, School of Medicine, University of Colorado Anschutz Medical Campus, Aurora, CO 80045, USA

**Keywords:** Zebrafish, Standards, Metadata

## Abstract

Zebrafish (*Danio rerio*) is a leading vertebrate model that has greatly advanced research across fields such as developmental biology, toxicology, immunology and genetics. The rapid generation of high-throughput datasets fueled by advances in genomics, imaging and artificial intelligence (AI) has expanded the zebrafish as an animal model for human disease research and therapeutic discoveries. However, the absence of globally adopted, standardized data reporting methods within the zebrafish community undermines data usability, interoperability and reproducibility. Inconsistent documentation of experimental parameters – including genes, alleles, developmental stages and imaging details – creates barriers to integrating and comparing results across laboratories and disciplines. Challenges are especially pronounced for reporting developmental stages, imaging metadata and chemical exposure protocols, impeding robust data integration and reuse. Although resources such as the Zebrafish Information Network (ZFIN) and global initiatives such as the Monarch Initiative promote ontology-driven data standards, widespread implementation remains limited owing to gaps in community awareness and engagement. The use of species-specific and integrative ontologies is essential for unambiguous data annotation and reliable cross-species comparison, particularly in the context of accelerating AI-driven research. In this Editorial, we summarize current standards relevant to the zebrafish field and highlight the urgent need for collective action. Broad community participation in developing, refining and consistently adopting robust data standards will enhance reproducibility, facilitate interdisciplinary collaboration and ensure that zebrafish research remains a pillar for future scientific and AI-powered advances.

## Teaser

**Summary:** This Editorial calls for active participation in data standardization for reporting and metadata integration to strengthen reproducibility, data integration and AI readiness for biomedical research discoveries and collaboration.

## Introduction

The ‘FAIR Guiding Principles for scientific data management and stewardship’, published in *Scientific Data* in 2016, intended to provide guidelines to improve the findability, accessibility, interoperability and reuse of digital assets (Wilkinson et al., 2016). The principles apply to three entities: data (or any digital object), metadata (information about that digital object) and infrastructure. The principles emphasize the capacity of computational systems to find, access, interoperate and reuse data with minimal or no human intervention. This is increasingly crucial because researchers rely on computational support to manage the growing volume, complexity and speed of data generation. With the advancement in cloud-based infrastructure and artificial intelligence (AI) tools, many scientific communities, regulatory agencies, funding agencies, journal publishers and other organizations have aligned with these principles.

Zebrafish as an animal model has enabled many scientific discoveries and supported multi-animal model studies, including in the context of human disease modeling (Cassar et al., 2020; Choi et al., 2021; Hill et al., 2005; Ochenkowska et al., 2022; Zhang and Zhou, 2024). Discoveries made by numerous independent teams, typically research laboratories, are commonly reported through scientific publications and datasets generated from high-throughput experiments. They are part of the general ‘body of knowledge’, which informs future research, enables cross-species comparison, such as those in the Monarch Initiative (Putman et al., 2024), and supports predictive tools like AlphaFold (Jumper et al., 2021; Varadi et al., 2022). With advances in genomics and imaging technologies, the combination of robust animal models for research and breakthroughs in AI enables researchers to constantly generate large datasets (Bobrovskikh et al., 2024; Gut et al., 2017; MacInnes, 2020; Unlu et al., 2019). Curating, integrating and interpreting such datasets is a challenge if there are no accepted and adopted standards: the information is often inconsistent and/or ambiguous, and it is unclear whether authors of different studies report the same entity (e.g. genes, alleles). A data standard is an agreed-upon method, definition or guideline for consistent quantification, measurement or description for exchanging data (Eunice Kennedy Shriver National Institute of Child Health and Human Development, 2024; Institute of Medicine (US) Committee on Data Standards for Patient Safety, 2004).

**Fig. 1.**
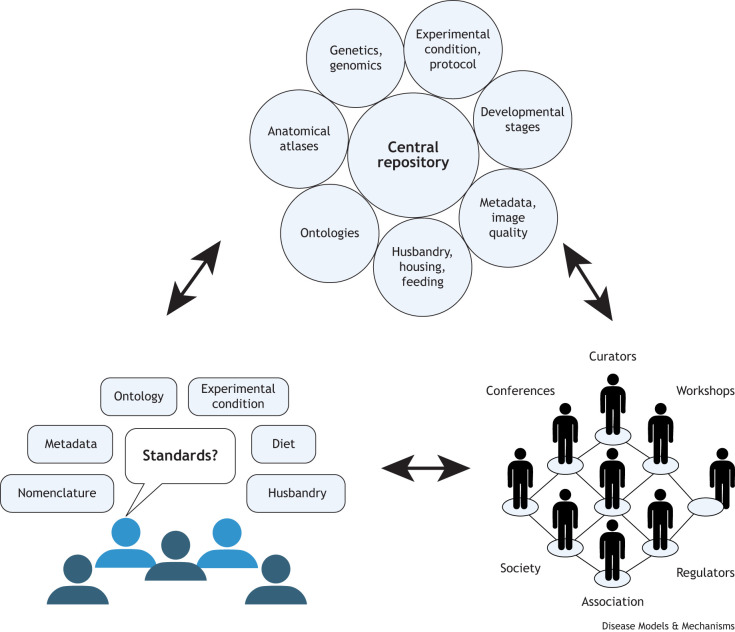
**Building a framework for reproducibility, standardized data reporting and metadata usage in zebrafish research.** A community-driven framework linking researchers, curators and organizations through a central repository that integrates zebrafish genetics, ontologies, experimental conditions, developmental stages, anatomical atlases, husbandry practices, metadata and image quality. Bidirectional feedback between contributors and standard developers, supported by workshops, conferences and regulatory bodies, drives consensus and standard adoption, ensuring interoperability, reproducibility and AI readiness of zebrafish research data.

Standards are fundamental to all research, including zebrafish studies, as they ensure data consistency, interoperability and usability across different systems, species, organizations and research fields (Wilkinson et al., 2016). They enable seamless communication and data sharing while maintaining data quality and reliability. This need for standards does not only apply to specific entities, but it also applies to the reporting of the minimal information for a research domain, ensuring data integration and reproducibility. The need to report standardized data has been an ongoing pursuit (Khan, 2017; McClary-Gutierrez et al., 2021). Effective metadata documentation in scientific manuscripts is also essential for the integrity and dissemination of research. However, the engagement of the zebrafish research community in adopting these standards is still lagging. To address this gap, we urge the zebrafish research community to participate in constant discussions to reach a consensus and adopt standards that should be made available through a central repository (Fig. 1).

### Standards for data disambiguation

Standards exist for most research domains in the form of agreed-upon, validated or established vocabularies or terminologies, taxonomies or ontologies, where each term is represented by a permanent and unique identifier (ID). By referring to entities such as genes, alleles and anatomies using a standard ID, we make these entities unambiguous and clearly identifiable, and related information referring to the same ID can then be confidently integrated and compared.

Within the zebrafish research community, the Zebrafish Information Network (ZFIN) serves as the central repository for zebrafish genetic and genomic data supporting information on mutants, transgenic lines and more (Bradford et al., 2017, 2023, 2025; Howe et al., 2021; Sprague et al., 2001). It curates anatomical, molecular and functional data using ontologies and standardized terminologies. They provide allele designation for fish lines, including mutants and transgenics. These allele designations (associated with the ID starting with ZDB-ALT-##) are required to include any allele-related information in the database. Similarly, genes are referred to by an ID from National Center for Biotechnology Information (NCBI), Ensembl or ZFIN (starting with ZDB-GENE-##) to unequivocally identify the allele or gene of interest using these standard IDs (Table 1).

**Table 1.** Existing standards for zebrafish data

Domain	Standard name	Example	Type	Browse terms	Repository	Reference
Allele	ZFIN line designation	*kc13* (ZDB-ALT-000831-6)	Identifier from ZFIN	N/A	https://zfin.org/action/feature/line-designations	atlassian.net
Gene	NCBI gene	*fgf8a* (NCBI gene ID 30538)	Identifier from NCBI	N/A	https://www.ncbi.nlm.nih.gov/gene/	N/A
	Ensembl gene	*fgf8a* (ENSDARG00000003399)	Identifier from Ensembl	N/A	https://www.ensembl.org/Danio_rerio/Info/Index	N/A
	ZFIN gene	*fgf8a* (ZDB-GENE-990415-72)	Identifier from ZFIN	N/A	https://zfin.org	N/A
Anatomy	Zebrafish anatomy and development ontology (ZFA)	Brain (ZFA:0000008)	Ontology	https://www.ebi.ac.uk/ols4/ontologies/zfa	https://github.com/ZFIN/zebrafish-anatomical-ontology	Van Slyke et al., 2014
Developmental stage	Zebrafish developmental stages ontology (ZFS)	Gastrula:bud (ZFS:0000022)	Ontology	https://www.ebi.ac.uk/ols4/ontologies/zfs	https://github.com/ZFIN/zebrafish-anatomical-ontology	Kimmel et al., 1995; Van Slyke et al., 2014
	Zebrafish postembryonic development	Squamation onset posterior	List of term		N/A	Parichy et al., 2009
Experimental condition	ZECO	Chemical treatment (ZECO:0000238)	Ontology	https://www.ebi.ac.uk/ols4/ontologies/zeco	https://github.com/ZFIN/zebrafish-experimental-conditions-ontology	Bradford et al., 2017

NCBI, National Center for Biotechnology Information; N/A, not applicable; ZECO, Zebrafish Experimental Conditions Ontology; ZFA, Zebrafish Anatomical Ontology; ZFIN, Zebrafish Information Network; ZFS, zebrafish developmental stage.

Ontologies provide structured representations of knowledge in which each concept is explicitly defined, enriched with metadata (e.g. synonyms) and linked to other terms through formal relationships that enhance context, interoperability and support for complex queries. For example, the Zebrafish Anatomical Ontology (ZFA) is a standard for zebrafish gross and cellular anatomy (Van Slyke et al., 2014) (Table 1). Notably, one of the main benefits of using ontology terms is that it enables cross-species comparison. For instance, Uber Anatomy Ontology (Uberon), a multi-species anatomy ontology, bridges different species-specific anatomy ontologies, including ZFA (Haendel et al., 2014). Its utility lies in providing a standardized vocabulary and hierarchical structure for anatomical concepts, enabling consistent annotation, integration and interpretation of heterogeneous biological data. Similarly, Unified Phenotype Ontology (uPheno) serves as an ontological bridge that unifies species-specific phenotype ontologies – such as the Human Phenotype Ontology (HPO), Mammalian Phenotype Ontology (MP), Zebrafish Phenotype Ontology (ZP) and others – into a single, semantically coherent system (Matentzoglu et al., 2025). Therefore, reporting zebrafish data using standard identifiers and ontologies not only supports unambiguity but also allows for comparison with other species.

Standards and ontologies are dynamic resources that evolve through continuous community input. Most are open source, allowing users to propose refinements and request new terms to meet emerging research needs. This collaborative process ensures that reporting with standardized IDs and terminologies remains current, relevant and extensible, while also facilitating the development of new standards when required.

### Standards for consistent anatomical framework

The development of standardized data collection and reporting practices in zebrafish research begins with establishing a consistent anatomical framework. One existing anatomy guideline is the zebrafish developmental staging series, which provides detailed descriptions and images of each embryonic stage (0-72 h) (Kimmel et al., 1995). Most zebrafish atlases are available online as open-access resources and sometimes hosted on databases (Table 2). A whole-zebrafish anatomical atlas across life stages annotated using anatomical ontology, such as ZFA, will provide a spatially resolved coordinate system that enables researchers to map, label and segment anatomical regions uniformly across studies. The Zebrafish Brain Atlas (Wullimann et al., 1996) has been instrumental in defining regions of interest across imaging modalities such as confocal microscopy, light-sheet microscopy and functional magnetic resonance imaging by standardizing the delineation of brain structures – such as the optic tectum, cerebellum or hypothalamus – using standard terms. The lack of an annotated unified anatomical atlas is a bottleneck; creating one would resolve long-standing challenges in cross-study comparisons of imaging data, eliminating ambiguity in data interpretation and facilitating cross-study comparisons.

**Table 2.** List of current web-based atlases related to zebrafish

Atlases	Stage	Attributes	Web address	Data type	Reference
3d.fish	2-5 dpf, 33 dpf	Microanatomy, microCT	http://3d.fish/	microCT	Ding et al., 2019
Bio-Atlas	48 hpf to 12 mpf	Microanatomy atlas, histology	https://bio-atlas.psu.edu/zf/index.php	Histology	Copper et al., 2018
Daniocell	3.3 hpf to 120 dpf	Single-cell atlas	https://daniocell.nichd.nih.gov/	Genomics	Farrell et al., 2018; Sur et al., 2023
FishFace		3D visualization, craniofacial development, tomography, confocal microscopy	https://www.facebase.org/resources/zebrafish/fishface/home/	microCT, confocal microscopy and more	Eames et al., 2013
MapZebrain	6 dpf	Brain atlas, neuron tracings	https://mapzebrain.org/home	RNA *in situ* labeling and antibody staining	Kunst et al., 2019; Shainer et al., 2023
Interactive Atlas of Zebrafish Vascular Anatomy	28 hpf to 7 dpf	Vascular anatomy, confocal microscopy	https://zfish.nichd.nih.gov/Intro%20Page/intro1.html	3D visualization and confocal microscopy	Isogai et al., 2001
Transgenic Zebrafish Brain Browser	6 dpf	Brain atlas, confocal, 3D visualization	http://vis.arc.vt.edu/projects/zbb/	Confocal microscopy	Marquart et al., 2015
Z Brain Atlas	6 dpf	Brain atlas, electron microscopy data, 3D visualization	https://zebrafishexplorer.zib.de/home/	Electron microscopy and light sheet	Randlett et al., 2015

dpf, days post-fertilization; hpf, hours post fertilization; microCT, micro-computed tomography; mpf, months post-fertilization.

### Key challenges in data reuse and reproducibility

Key factors that impact data reuse and reproducibility but are often overlooked include metadata completeness and image quality in scientific reporting. Metadata are data about data: they provide descriptive, structural and administrative information that help contextualize, interpret, manage and reuse data effectively. They are essential for ensuring data transparency, reproducibility and interoperability, particularly in large-scale, complex datasets generated through genomics, imaging or toxicological assays.

Zebrafish husbandry practices differ considerably across facilities. Although most protocols are adequate to maintain zebrafish welfare and physiology, experimental reproducibility can be substantially improved through standardized husbandry guidelines. Zebrafish husbandry parameters, transgenic line identifiers or developmental staging ontologies are not easily found in most publications. Key factors that require standardization include housing conditions (e.g. water chemistry, light cycle, temperature), diet and feeding, and reproduction and breeding (Aleström et al., 2020; Lawrence, 2007; Lawrence and Mason, 2012; Licitra et al., 2024; Watts and D'Abramo, 2021). Establishing zebrafish-specific husbandry practices, integrated with central resources such as ZFIN, would provide a structured platform to promote and disseminate these metadata. By aligning with global data archives while maintaining zebrafish-specific fidelity, such a layered approach would ensure that metadata remain findable, accessible, interoperable and reusable (FAIR), as well as cross-compatible, thereby enhancing rigor and reproducibility in zebrafish research.

Zebrafish research often integrates multiple imaging modalities, such as brightfield microscopy, fluorescence imaging and molecular data. Inconsistencies in imaging metadata remain a major barrier; identical fluorescent reporter lines imaged at different magnifications and orientations are difficult to integrate across studies. As research tools and techniques rapidly evolve, the scientific community must continuously update standards to integrate new technologies and methodologies. Although these tools enable researchers to gather unprecedented amounts of data, they also introduce complexity, variability and the potential for inconsistent data formats if not standardized (Schadt et al., 2010). One such database is MapZebrain, an open-access, integrative digital atlas platform for the 6-days post-fertilization zebrafish brain, designed to provide a high-resolution, standardized spatial framework for mapping neuronal circuits, gene expression and cellular data (Kunst et al., 2019; Shainer et al., 2023; Svara et al., 2022). It was developed to overcome the challenge of comparing and integrating data across studies, which often vary in anatomical annotations, orientation and resolution. It supports 3D visualization and multimodal data integration, making it a valuable resource for researchers in neurobiology, developmental biology and systems neuroscience. If scale bars, orientation conventions and acquisition parameters were reported in a standardized way, images could readily be aligned to resources such as MapZebrain or the developing unified anatomical atlas.

A unified platform for zebrafish research is crucial, as this model is widely utilized in fields ranging from developmental biology and genetics to toxicology, neuroscience and regenerative medicine. Differences in experimental protocol between laboratories significantly affect how chemical-mediated effects are reported and hinder reproducibility (Hsieh et al., 2023). To overcome these challenges, standardized guidelines must be developed for experimental protocols and metadata required for reporting. Key inconsistencies in reporting zebrafish exposure results or toxicology data include measurement units of chemical concentration (mg/l versus nM or mM), exposure duration and time points (hours versus days), and exposure endpoints. Toxicology assays often report concentrations in incompatible units (mg/l versus molarity) or vary in describing exposure endpoints (e.g. mortality, morphological defects, behavioral measures).

Similarly, single-cell transcriptomic datasets in zebrafish vary widely in their annotation practices, with some studies mapping cells to developmental stages using morphology-based timing, while others use bulk RNA-sequencing markers (Vöcking and Famulski, 2023). This inconsistency hampers cross-study comparison and undermines integration into global atlases. A zebrafish-specific metadata standard for reporting transcriptomic data would ensure that cell-type annotations and developmental staging are harmonized, facilitating direct comparisons with mouse or human datasets. Without harmonization, integrating or meta-analyzing results across laboratories remains nearly impossible. Implementing robust, standardized metadata frameworks similar to the ‘minimum information about a microarray experiment’ (MIAME) standards would support reproducibility and facilitate predictive modeling relevant to human health.

### Roles of the scientific community in developing and adopting standards

Developing and ensuring adherence to research standards remains challenging owing to various factors, including technical limitations, lack of specialized training and resistance to change. Addressing these challenges requires a coordinated effort from the scientific community, with community-driven engagement playing a vital role in enhancing zebrafish research reproducibility.

### Community-driven consensus building

The scientific community's input is essential for developing and refining standards through consensus-driven processes. Workshops, conferences and professional organizations such as the International Organization for Standardization (ISO) or field-specific groups like the International Zebrafish Society (IZFS), Zebrafish Disease Models Society (ZDMS) and Zebrafish Husbandry Association (ZHA) provide platforms for discussions and fostering consensus, such as guidelines for euthanasia (AVMA Guidelines for the Euthanasia of Animals, 2020; Matthews and Varga, 2012). Through such gatherings, scientists can collaboratively address challenges, propose updates and establish standards that align with the community's evolving needs. A historical example of such consensus building is the establishment of zebrafish nomenclature conventions widely adopted by the zebrafish research community. The conventions are updates to rules established during a discussion session at a meeting in Rinberg, Germany, in 1992 (Westerfield, 2000). ZHA has initiated the ‘Zebrafish Husbandry Reporting and Reproducibility Initiative’ to develop a minimal reporting checklist, including clear guidelines for key environmental and care parameters to be consistently included in zebrafish scientific publications.

In addition to fostering an environment of continuous dialogue and collaboration, a central repository for standards is needed as a concerted effort from the scientific community to effectively develop and sustain standards in enhancing zebrafish research reproducibility and interoperability. The Monarch Initiative has demonstrated that data standardization efforts are critical for advancing rare disease diagnostics, functional genomics and systems biology. Community consensus of having a central repository for zebrafish data, data standards and references will enable ease of access and encourage participation of the community.

### Incorporating feedback from users and researchers

The practical implementation of standards often reveals limitations or areas for improvement that may not have been apparent in theory. Researchers working hands-on with data and standards in their studies provide invaluable feedback that drives iterative refinements to accommodate new research questions, contexts, or technologies (Goodman et al., 2016). The iterative versioning process is fundamental for allowing standards to evolve incrementally. During the era of -omics advancement, continuous updates to formats like FASTQ for sequencing data or the development of variant call format (VCF) exemplify how community feedback has refined these standards over time, making them more versatile and applicable to a broader range of research applications (Danecek et al., 2011). In keeping with this approach, active input from organizations such as IZFS, ZFIN and broader scientific communities will be crucial in incorporating and refining the agreed-upon standard framework for implementation.

### AI readiness

AI is making a growing impact on zebrafish research. AI's ability to accelerate and enhance the analysis of large datasets, automate tasks and improve the accuracy and efficiency of research has allowed its application in various fields such as behavior analysis, genomics, neuroscience, disease modeling and more. AI thrives on large-scale, high-quality data. Standardized data formats and ontologies allow seamless integration of datasets from diverse sources, enabling AI models to learn from broader and diverse information, validate findings, compare results across studies and minimize errors caused by inconsistencies.

To fully realize the potential of AI in scientific discovery and innovation, it is essential that the zebrafish research community actively engage in data standardization and data reporting. Clear reporting standards ensure that datasets are representative and well documented, reducing biases in AI models. When metadata, provenance and methodologies are standardized, AI can better account for potential biases, ensuring fairer and more ethical decision-making. Ontologies help AI make meaningful connections between different research fields. Future AI systems will rely on decades of accumulated data. Standards ensure that past research remains accessible, interpretable and valuable for AI-driven insights, preventing knowledge loss due to outdated formats or ambiguous reporting practices. By adopting consistent formats, ontologies and clear reporting standards, zebrafish data can be easily accessible, interpretable and valuable for AI-driven insights. As AI becomes more central to scientific progress, these standards will ensure reliable, reproducible and impactful discoveries.

## Call to action and summary

To fully realize the potential of zebrafish as a model for scientific discovery and biomedical advancement, the zebrafish research community must move decisively toward widespread, coordinated adoption of data and metadata standards. The urgency of this task is underscored by the expanding scale and diversity of datasets**,** particularly spatial and single-cell sequencing, imaging and high-throughput chemical screening, and by the growing opportunities presented by AI for cross-study integration and new knowledge generation. With AI poised to transform biological discovery, well-structured zebrafish-specific datasets will ensure that decades of research remain interpretable, reusable and impactful. Standardization is no longer optional; it is the prerequisite for reproducibility, interoperability and meaningful AI-driven insights.

We propose three specific priorities for the zebrafish research community:

(1) **Develop a centralized metadata checklist for zebrafish research**, covering domains such as husbandry parameters, developmental staging, genotype nomenclature, chemical treatment protocols and imaging methods. This checklist should be a prerequisite for publication and data submission and should be regularly reviewed and updated by the community.
(2) **Establish and maintain a unified zebrafish anatomical atlas**, annotated throughout embryonic and post-embryonic stages with community-agreed ontologies (e.g. ZFA, ZFS). This resource should be openly accessible and actively curated, providing a gold standard for spatial data annotation and facilitating alignment across imaging and single-cell studies.
(3) **Invest and participate in the review and expansion of data repositories and standards portals.** Platforms such as ZFIN and the Monarch Initiative leverage centralized standards (including ontologies) to integrate heterogeneous datasets, creating robust infrastructures for the storage, annotation and dissemination of richly curated, interoperable data that drive large-scale discovery and translational research. The zebrafish community should play an active role in this effort by sharing expertise and critically reviewing standards to ensure accuracy and consistency. Close collaboration with the biocurator community is particularly important to guarantee proper application of these standards. To further promote adoption, community meetings should incorporate presentations and hands-on workshops focused on standards. Such engagement will substantially increase the likelihood of widespread standard adoption, ultimately enhancing data integration, reusability and readiness for AI-driven analyses.

Sustained progress will require regular, community-driven workshops and targeted working groups at scientific meetings to discuss, test and adopt new and refined standards as technologies, data types and research questions evolve. With these steps, the zebrafish community will not only enhance reproducibility and interoperability within the field but will also ensure that zebrafish research remains at the forefront of integrative, AI-powered biological discovery for years to come.
